# Complete Mitochondrial Genome and Phylogeny of Pleistocene MammothMammuthus primigenius

**DOI:** 10.1371/journal.pbio.0040073

**Published:** 2006-02-07

**Authors:** Evgeny I Rogaev, Yuri K Moliaka, Boris A Malyarchuk, Fyodor A Kondrashov, Miroslava V Derenko, Ilya Chumakov, Anastasia P Grigorenko

**Affiliations:** **1**Brudnick Neuropsychiatric Research Institute, Department of Psychiatry, University of Massachusetts Medical School, Worcester, Massachusetts, United States of America; **2**Laboratory of Molecular Brain Genetics, Research Center of Mental Health, Russian Academy of Medical Sciences, Moscow, Russia; **3**Faculty of Bioengineering and Bioinformatics, Lomonosov Moscow State University, Moscow, Russia; **4**Vavilov Institute of General Genetics, Russian Academy of Sciences, Moscow, Russia; **5**Research Center of Medical Genetics, Russian Academy of Medical Sciences, Moscow, Russia; **6**Genetics Laboratory, Institute of Biological Problems of the North, Russian Academy of Sciences, Magadan, Russia; **7**Section of Ecology, Behavior and Evolution, University of California San Diego, La Jolla, California, United States of America; **8**Serono Genetics Institute SA, Evry Cedex, France; Massey UniversityNew Zealand

## Abstract

Phylogenetic relationships between the extinct woolly mammoth*(Mammuthus primigenius),* and the Asian*(Elephas maximus)* and African savanna*(Loxodonta africana)* elephants remain unresolved. Here, we report the sequence of the complete mitochondrial genome (16,842 base pairs) of a woolly mammoth extracted from permafrost-preserved remains from the Pleistocene epoch—the oldest mitochondrial genome sequence determined to date. We demonstrate that well-preserved mitochondrial genome fragments, as long as ~1,600–1700 base pairs, can be retrieved from pre-Holocene remains of an extinct species. Phylogenetic reconstruction of the Elephantinae clade suggests thatM. primigenius andE. maximus are sister species that diverged soon after their common ancestor split from theL. africana lineage. Low nucleotide diversity found between independently determined mitochondrial genomic sequences of woolly mammoths separated geographically and in time suggests that north-eastern Siberia was occupied by a relatively homogeneous population ofM. primigenius throughout the late Pleistocene.

## Abstract 2

The complete mitochondrial genome of a 33,000-year-old Mammoth confirms its close relationship with the Asian elephant and suggests that genetic diversity among mammoths in Siberia during the late Pleistocene was low

## Introduction

*Mammuthus, Elephas,* and*Loxodonta* (family Elephantidae, subfamily Elephantinae) are closely related genera that evolved in the African Pliocene, possibly from the genus*Primelephas* [1–4]. The woolly mammothMammuthus primigenius became extinct across most of its former range along with other late Pleistocene megafauna, although small, isolated mammoth populations survived into the mid-Holocene [5]. The phylogeny of Elephantinae has not been resolved. Morphological analyses have yielded conflicting phylogenies for*M. primigenius, Elephas maximus,* andLoxodonta africana [1–4]. Dental characters suggest a closer relationship betweenM. primigenius and*E. maximus,* trunk tip morphology supports a grouping ofM. primigenius and*L. africana,* and immunological and hair structure characters could not confidently resolve the phylogeny of these three taxa [1–4]. Molecular analyses have also generated conflicting conclusions [1–4]. Data on short mitochondrial DNA (mtDNA) sequences have variously supported a monophyletic clade of the extant elephant species, have groupedM. primigenius with either*E. maximus* [6] orL. africana [7–9], or have been inconclusive [10,11]. DNA template modifications caused by oxidation or hydrolysis are a potential source of artificial mutations and may partly explain the high polymorphism initially observed for some short mammoth DNA sequences, although pseudogenes of mitochondrial origin located in the nuclear genome, which are common in elephants, may also be a potential factor [10]. A meta-analysis taking into account potential sequencing and other errors favored the*M. primigenius–L. africana* clade [9]. Clearly, a comprehensive analysis of longer genomic sequences is necessary to resolve this phylogeny, but so far only short DNA fragments have been retrieved fromM. primigenius.

## Results/Discussion

**Figure 1:**
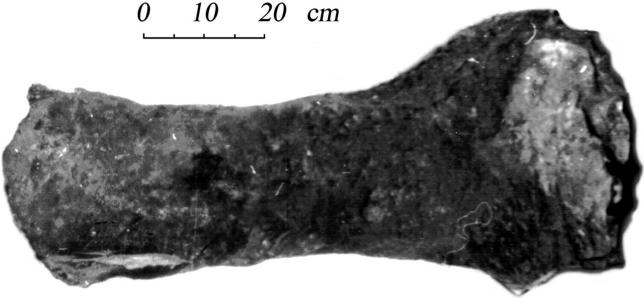
The Right Back Leg of the Woolly Mammoth*(M. primigenius)* Found in Siberia The well-preserved mammoth body fragment with foot (33 × 36 cm), shin, and ankle-joint (the total length is ~88 cm) was found in the Enmynveem River valley (north-eastern Siberia, Chukotka). The tissue material (bones, muscles, and skin) had no visible marks of tissue damage by insects or other animals. Radiocarbon dating of the skin and muscle tissue determined that the mammoth lived 32,850 ± 900 y ago [12].

**Figure 2:**
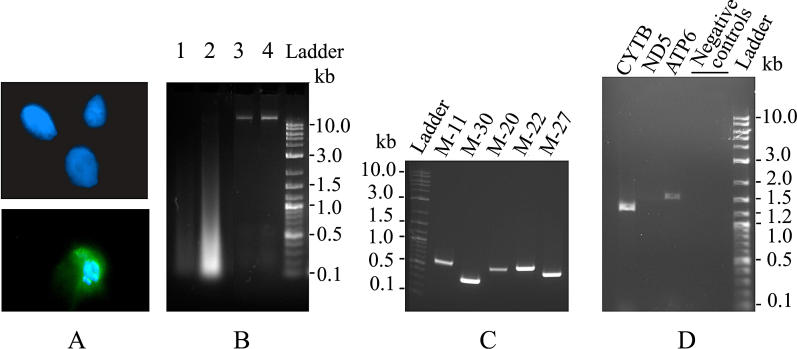
The Unusually Well-Preserved Mammoth DNA (A) Nuclei with DNA clearly detectable by DAPI staining in muscle cells of ~33,000-y-oldM. primigenius. (B) Total genomic DNA isolated from the mammoth muscle tissue (lane 1 is 1/10 dilution of the DNA on lane 2); control DNA from fresh human blood samples in lanes 3 and 4. (C) Examples of PCR products (~300–600 bp) for mammoth mitochondrial genome. (D) PCR amplification recovers long sequences for complete mitochondrial genes (1,317 bp*CytB* and 1,613 bp*ATP6* genes), but PCR of larger fragments (3,054 bp*ND5*) is failed.

The woolly mammoth leg with intact muscle and skin tissue was found in the Enmynveem River valley (Chukotka) in north-eastern Siberia in 1986 (Figures 1 andS1). Since then, the collected muscle specimens of the mammoth (hereafter called Enmyn) were kept frozen. Radiocarbon method dated the specimens to ~33,750–31,950 y BP (before present) [12]. An initial examination suggested that the soft tissue was remarkably well preserved, and no signs of tissue decay were noticed when the specimen was excavated from the permafrost. Because no tissue damage by insects or other animals was observed, the remains presumably were quickly buried and have never been defrosted. The cells with nuclei were observed in epithelia and muscle tissue. The treatment with DAPI efficiently stained the nuclei, indicating the existence of well-preserved genomic DNA (Figure 2A). To our knowledge, this is the first documented cytogenetically detectable nucleus with genomic DNA in such an ancient tissue (>30,000 y BP).

In accordance with these primary observations, a substantial amount of genomic DNA was extracted and detected on an electrophoresis gel. Although the extracted DNA was apparently degraded, it is yet of remarkable quality and quantity. For example, the major fraction of DNA fragments from one extract (shown inFigure 2B) ranged from 100 base pairs (bp) to 600 bp, with diminishing amounts at higher molecular weights. Microbial DNA may potentially contribute to the high-molecular-weight DNA fraction. Nevertheless, the PCR analysis described below (Figure 2C and2D) supports the assertion that this mammoth specimen contains preserved DNA. Independent replication in PCR analysis is absolutely imperative for the study of ancient DNA. Therefore, several DNA extracts from the tissue specimens were obtained for the mitochondrial genome analysis.

Importantly, the replication in this study was not limited to selected mitochondrial genome regions, and the entire mitochondrial genome was sequenced in duplicate. Different DNA extracts from mammoth muscle tissue were used to generate PCR products and reconstruct the complete mitochondrial genome in Moscow (“MOS contig”, completed in 2000) and, independently, at the University of Massachusetts Medical School (UMASS MS) laboratories (“UM contig”, completed in 2005) (seeMaterials and Methods). No contamination by extraneous DNA was found in multiple PCR experiments. Apart from a variable number of tandem repeats (VNTR) in the control region characterized by high somatic hypervariability, the mtDNA sequences obtained from different extracts and different laboratories matched exactly. This accuracy was achieved with the following protocol. (I) Redundant oligonucleotide primers were designed and tested to yield a sufficient amount of PCR products (Figures S2–S4 andTable S1). Preliminary tests with these primers were conducted to demonstrate the applicability of DNA extracts for PCR. We consistently found efficient recovery of at least ~500- to 700-bp PCR fragments from mammoth DNA extracts. Moreover, PCR of complete gene sequences of ~1,200–1,700 bp was also efficient, but the mtDNA sequence larger than 3,000 bp was not amplifiable (Figure 2C and2D and Methods and Materials). The data are consistent with excellent preservation coupled with some degradation due to the ancient origin of the DNA. (II) The large amount of DNA enabled us to obtain PCR products sufficient for sequencing after the first round of PCR reaction and minimized the risk that sequence errors arose due to template switching during PCR, because of the low number of original DNA templates [13,14]. (III) Direct sequencing of PCR products gave clean chromatogram reads (Figure S5). In all cases, the replication of sequencing in each nucleotide position was performed from different PCR products using the protocols described inMaterials and Methods. In addition, the PCR products were cloned and the independent clones were also sequenced. As expected, the cloned mtDNA fragments occasionally contained random mutations with an average rate of ~6/1,000 bp. No cloned sequences with identical sets of mutations were identified. These sequence modifications were mostly transitions corresponding to type I (A→G/T→C) and type II (C→T/G→A) mutations, which have previously been detected in ancient DNA (Figure S6) [13–15]. As described previously for ancient DNA, type II mutations were predominant in the cloned mammoth mtDNA sequences (>70%). The sequence modifications were not detectable in direct PCR product sequences of the same region (Figure S5). The data indicate that the mutations in cloned DNA were random and rare in each site of individual molecules. No contamination with nuclear mitochondrial DNA (numt) was found for any PCR product of mammoth DNA (Materials and Methods). Direct sequencing of multiple PCR products and independent clones provided evidence of accurate reconstruction of authentic mitochondrial genome sequence ofM. primigenius.

The sequence of the small region (VNTR) in the control region (D-loop) could not be determined by direct sequencing of PCR products. Thus, the analysis of cloned PCR fragments was undertaken. The sequence analysis demonstrated somatic heteroplasmy and high molecular heterogeneity in this region. The heterogeneity resulted from hypervariability in a number of short hexa-nucleotide tandem repeats (CGCATA)_n_ resembling VNTR (Figure S4). Similar VNTR was found in the control mtDNA region in*Loxodonta* and*Elephas* also.

To determine the evolutionary history of the woolly mammoth, we sequenced complete mitochondrial genomes ofL. africana andE. maximus. To exclude the possibility of DNA contamination, the elephant specimens were obtained only after the primary mitochondrial genome sequence ofM. primigenius was completed (Materials and Methods). Long-range PCR was initially used for the amplification of the overlapping ~3–5 kb mtDNA fragments of African and Asian elephants. The sequence analysis was also conducted for cloned PCR fragments and short PCR fragments as described in mammoth mtDNA analysis. TheE. maximus andL. africana mitochondrial genomes sequenced in this study were highly similar but not identical to those submitted to GenBank previously.

**Figure 3:**
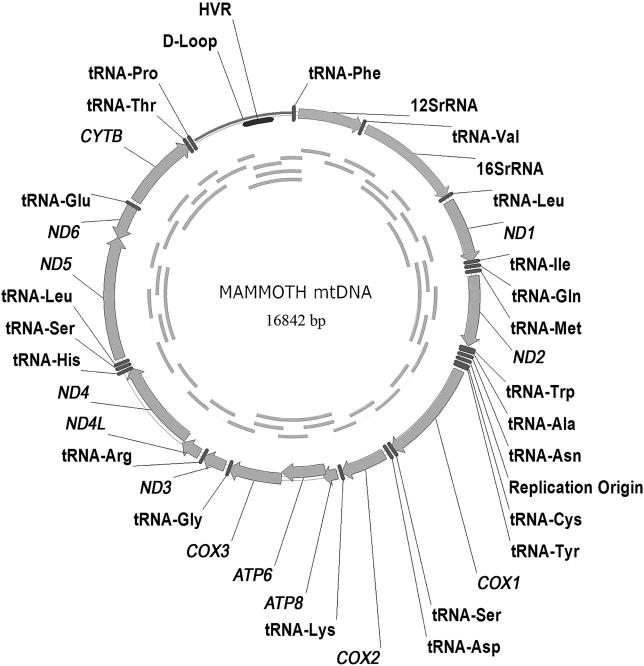
Mitochondrial Genome of the Woolly MammothM. primigenius The complete mitochondrial genome was determined independently in two different laboratories using designs with multiple primers for overlapping PCR fragments ranged from ~325 to ~650 bp; the longer PCR fragments were also produced and sequenced. The overlapped PCR products used for sequencing and cloning are shown by the inner circle. Only PCR fragments produced from different pairs of primers are shown. Two genes,*ATP6* and*ND4L,* overlap with neighboring genes.

Similar to other placental mammals, the mammoth mitochondrial genome contains 13 protein coding genes, 22 tRNA and two rRNA genes, and the D-loop control region (Figure 3). The length of the genome varied due to somatic variability of tandem DNA repeats (CGCATA)_n_ in the control region. The length of the mammoth mitochondrial genome was 16,842 bp, if the longest VNTR tract was included (Figure S4). Start and stop codons were identical in*M. primigenius, L. africana,* and*E. maximus;* however, we found a substitution (G→A) in the*ND4* gene leading to a premature stop codon in the C-terminus of the protein. Thus, the ND4 protein is four amino acids shorter inM. primigenius than in elephants. A multiple alignment of this gene showed that the C-terminal end of the ND4 protein is variable in sequence and in length in other animal species. Substitutions in the mammoth lineage were predominantly transitions (19/339 transversion/transition ratio in mammoth versus 13/351 inE. maximus when polarized withL. africana). The total number of nucleotide and amino acid differences between the mammoth andE. maximus genomes was 722 and 100, and between the mammoth andL. africana genomes 780 and 107, respectively (Table S2).

**Figure 4:**
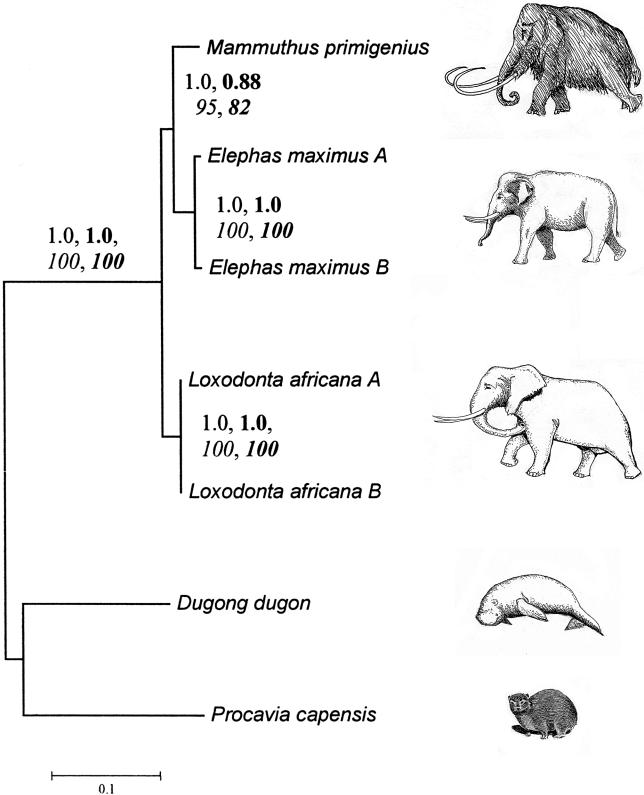
Paenungulata Tree and Phylogenic Relationship of the Woolly Mammoth The analysis of complete mtDNA sequences placesM. primigenius withE. maximus on the tree. The Sirenia*(D. dugon)* and Hyracoidea*(P. capensis),* most closely related species among extant taxa to Elephantinae, were taken as outgroups. Bootstrap values and posterior probabilities were calculated using a Bayesian approach [29,31] assuming a gamma distribution of the rates of evolution across sites with a General Time Reversible model (normal font), HKY model (bold), with a parsimony approach (italic), and by neighbor joining (italic and bold) [28]. The scale is 0.1 substitutions per site. The mitochondrial genomes of*M. primigenius, E. maximus A, E. maximus B, L. africana A, L. africana B, D. dugon,* andP. capensis were used in the analysis.

The phylogeny of Elephantinae was inferred using the newly obtained*M. primigenius, E. maximus,* andL. africana sequences, and other complete mitochondrial genome sequences ofE. maximus and*L. africana,* while the mitochondrial genomes of the closest extant taxa [16], the dugong*(Dugong dugon)* and the hyrax*(Procavia capensis),* were used as outgroups. The same topology was recovered by a variety of tree reconstruction methods (Figure 4). As expected, individuals from the same species,E. maximus and*L. africana,* clustered together on the tree.M. primigenius was determined to be a sister species to*E. maximus,* i.e., the woolly mammoth shared a common ancestor with the Asian elephant more recently than with the African elephant. A maximum likelihood (ML) ratio test comparing all three possible topologies of the Elephantinae species corroborates this conclusion (*p* < 0.01; seeMaterials and Methods). We also reconstructed the phylogeny of these species by using only individual protein and rRNA genes (tRNA genes are too short and contain too few substitutions). The majority, but not all (Table S3), of trees reconstructed with the sequence of individual genes supported the topology recovered using the complete genome. Although the topology of this tree appears robust, the unavailability of an outgroup more closely related than the dugong and hyrax calls for the sequencing of nuclearM. primigenius genes to further test the results reported here.

The resolution of the Elephantinae phylogeny makes it possible to estimate the time of divergence ofM. primigenius from its sister speciesE. maximus. Such an estimate usually relies on the existence of a molecular clock, i.e., a uniform rate of evolution of all compared clades, and on having calibration points from the fossil record. However, previous studies suggested that the evolution of the Elephantinae mitochondrial genomes may be inconsistent with the molecular clock [3,8], and so we tested this possibility using complete genomes. Tajima's relative rate test showed no difference in the rate of evolution of different Elephantinae lineages (unpublished data). Similarly, a likelihood ratio test did not reject the assumption of equal rate of evolution of theM. primigenius versus theE. maximus lineage using theL. africana genome as an outgroup (Materials and Methods). In contrast, the assumption of a molecular clock was rejected when the likelihood ratio test was performed on simulations involving a comparison of theM. primigenius–E. maximus clade with theL. africana lineage when the dugong and hyrax species were used as outgroups (without molecular clock LnL1 = −56661.45; with molecular clock LnL2 = −56877.63;*p* ≪ 0.01).

Molecular clock analyses are highly sensitive to the molecular distance of the outgroups, which can affect the correct root placement. Clearly, the dugong and hyrax are not ideal outgroups for this analyses, as their approximate nonsynonymous nucleotide divergence (dn) with the Elephantinae species is ~0.15 while synonymous nucleotide divergence is at saturation (ds ~ 1.2). Thus, the change in the rate of evolution implied by the ML analysis must be treated with caution. However, a simple parsimony assay corroborated the possibility that the mitochondrial genomes of different Elephantinae species evolve at different rates (Table S4). A ML estimate of the number of substitutions per lineage yielded quantitatively similar results (unpublished data).

These observations, coupled with previous reports [3,8], imply that a molecular clock assumption may be inappropriate when estimating the time of divergence of Elephantinae species. Thus, we used a heuristic rate smoothing procedure for ML estimates [17], which takes into account different rates of evolution among different branches of the tree. Using the calibration points of 6 million y for theE. maximus–L. africana split and 65 million y for the Elephantidae–Sirenia split (see [8] and references within), the time of divergence ofM. primigenius andE. maximus was estimated as ~4 million y ago (±0.01 s.e.). This estimate must be treated with caution because it relies on the correct paleontological dating of the*E. maximus–L. africana* divergence. Nevertheless, these data clearly indicate that the divergence of the woolly mammoth from the Asian elephant occurred soon after the divergence of their ancestor lineage from African elephants.

To further verify the accuracy of our sequence and to investigate the level of nucleotide diversity in the woolly mammoth population, we compared our sequence to the longest available sequences of the mammoth mitochondrial genome from other individuals. Two*CytB* gene sequences from mammoth individuals found in north-eastern Siberia (Magadan region, Kirgilyakh Creek near Kolima River) [18] and north-central Siberia (Pyasina River valley in Taimyr Peninsula) [19] show very high similarity, while the 12S RNA gene [19] was identical to the corresponding genes in our mitochondrial genome sequence from Chukotka's mammoth (Table S5). All these sequences were recovered from muscle specimens enriched in mtDNA and, thus, very likely had no numt contamination. The Chukotka Enmynveem River valley is ~1,000 km from Kirgilyakh Creek and ~2,900 km from the Pyasina River valley. The data suggest a relatively low genetic diversity of mammoth maternal lineages in mammoth population spanning vast territory in Northern Siberia. Two recent studies have also demonstrated the isolation of multiple fragments of mammoth DNA [20,21]. In one report, a novel approach was applied for direct cloning and shotgun sequencing of random DNA fragments. The set of multiple fragments corresponding to partial mitochondrial genome sequence contained sequences with ancient DNA artifact mutations and potential numts and cannot yet be reliably used for comparative analysis of complete mitochondrial genomes [21]. Another group reported the complete mitochondrial genome sequence [20] of a ~12,170-y-old woolly mammoth that was found in the Berelyekh Yakutia region (70° 35′ N, 145° 00′ E), which is ~900 km from the Enmynveem River valley (68° 10′ N, 165° 56′ E). A comparison of the two complete genomes revealed a pattern of high similarity with the nucleotide diversity of ~0.3% (Table S5). This diversity may be even lower since sequence errors may be present in the genome sequences recovered from ancient specimens. When the differences between the two mammoth mitochondrial genomes were polarized withE. maximus sequence, the number of sequence-specific polymorphisms for the mitochondrial genome sequence determined here was slightly lower (by ~25%) than in the mitochondrial genome sequence described in the other study [20]. The sequence [20] also showed a higher ratio of nonsynonymous to synonymous substitutions in protein coding genes and a higher number of nucleotide substitutions in the entire genome (C→T/G→A transitions) that are often associated with ancient DNA modifications (Tables S6 andS7). Although these differences were not statistically significant, a large number of nonsynonymous substitutions was observed in the*ND2* gene in sequence [20], but not in our sequence (Table S6). The possibility that these changes appeared due to positive selection in one of the mammoth lineages seems highly unlikely, the more reasonable explanation being that the genome sequence from [20] may harbor some post-mortem ancient DNA mutations (most*ND2* nonsynonymous mutations were C→T/G→A). However, these artifacts appear to be rare. In sum, the independently determined mitochondrial genome sequences from ~33,000-y-old (present study) and ~12,000-y-old [20] animals were highly similar, and, owing to a few potential errors, the true level of mitochondrial nucleotide variability may be slightly lower than 0.3%.

The low nucleotide diversity of mammoth mitochondrial sequence (π ~ 0.003;Table S5) is an order of magnitude lower than that reported for the overall populations ofL. africana (π ~ 0.02) [22] andE. maximus (π ~ 0.017) [23,24], but similar to the values reported for select populations ofL. africana (π ~ 0.00084–0.027) andE. maximus (π ~ 0.0024–0.0055) [22–24]. These data suggest that unlike the Asian and African elephants, the mammoth population has not had a complex population structure and has had a relatively low genetic diversity in mitochondrial lineages, at least in the area spanning thousands of kilometers in north-eastern Siberia. Further sequencing of mitochondrial genomes of other mammoth specimens can clarify the diversity of the ancient mammoth population.

The phylogenic reconstructions based on complete mitochondrial sequence analysis is a powerful method to determine the relationship between taxa or even closely related extinct and extant species as demonstrated here and elsewhere [25]. However, mitochondrial and nuclear DNA may have a different coalescent history, and cytonuclear-genomic dissociation has been described recently in African forest and savanna elephants [26]. Given the unique quality of the specimens from the Enmyn mammoth, nuclear DNA may potentially be recovered [21] and used for further confirmation of the topology reconstructed in this study. Our data demonstrate that very high-quality genomic DNA may be recovered from ancient remains of the Pleistocene age and that sequencing of complete mitochondrial genomes can lead to reliable phylogenic reconstructions and population studies not only for extant, but also for extinct species.

## Materials and Methods

### Extraction of genomic DNA

Microscopy analysis and DAPI staining of cells demonstrated the preservation of DNA in the mammoth specimens. Genomic DNA was extracted from the muscle mammoth specimens using silica-based and phenol-chloroform extraction methods, but the phenol-chloroform method was more efficient when extracting large amounts of DNA. The following protocol was used for phenol-chloroform DNA isolation. DNA was extracted from ~0.1–0.4 g of muscle tissue in a solution containing 50mM Tris-HCl (pH 8.0), 100mM NaCl, 10–25 mM EDTA, 1%–2% SDS, and at least 100 μg/ml proteinase K for 16–24 h at 37 °C, followed by phenol-chloroform extraction. DNA purification was completed by precipitation with 75% ethanol or by concentrating with Centricon YM centrifugal filter devices (with Amicon filter). DNA quality was analyzed by agarose electrophoresis and PCR.

### Mammoth DNA

First, DNA prepared from different samples and by different methods (silica-based and phenol-chloroform extraction methods) was used for PCR of the*CytB* gene. The analysis demonstrated that the sequences from various DNA extracts were identical and corresponded to previously reported short sequences for mammoth*CytB* gene. After the preliminary experiments, an average length of PCR product of 500- to 600-bp range was chosen to determine the complete mtDNA sequence ofM. primigenius. To design the PCR primers, we used the sequences of mitochondrial genome in the GenBank database available at the time. (Note: this project was launched in January 2000). Each primer pair was placed over the alignment of mitochondrial genomes of*L. africana,* hippopotamus, rhinoceros, and donkey. We gave preference to the primers located in the most conservative regions to keep the number of ambiguous nucleotides in designed oligonucleotide primers as small as possible. Ultimately, to amplify the whole mitochondrial genome of the mammoth, 35 pairs of redundant primers producing overlapping PCR fragments were designed.

On average, the length of overlapped sequences between PCR fragments produced from different primers was ~30 bp (excluding primer sequences), but were as long as ~200 bp in some cases. The anticipated size of the PCR fragments ranged from 325 bp to 650 bp and was 929 bp for the VNTR region. To determine the complete mtDNA sequence in UMASS MS, additional primer oligonucleotides were designed as shown inTable S1. On average, the mtDNA sequence was covered ~ nine times in MOS contig and ~ five times in UM contig by sequencing from different PCR products and clones. The total number of sequenced nucleotides determined from two complementary strands was 240,094. The total length of overlapped sequence obtained from different primers was at least 7,000 bp. In addition, we were able to produce several large PCR fragments (the sequence length between direct and reverse primers and corresponding mitochondrial genome region are indicated): L4-H5 968 bp (16S rRNA), L6-H7 1,061 bp (16S rRNA; tRNA Leu;*ND1*), L8-H10 1,642 bp (or 1,683 bp with primers) (*ND1;* tRNAs Ile, Gln, Met;*ND2;* tRNAs Trp, Ala, Asn), L16-H18 1,572 bp (*COX2;* tRNA Lys;*ATP8; ATP6; COX3*), L24-H26 1,502 bp (tRNA Leu;*ND5*), L29-H31 1,271 bp (tRNA Glu;*CYTB;* tRNAs Thr, Pro), L33-H35 1,131 bp (D-loop; tRNA Phe; 12S rRNA) that covered the shorter PCR fragments. No mismatches in the overlapped sequences or in the independent PCR products were found.

The standard PCR was conducted in total volume of 25 μl containing 1–2 U Taq1 DNA polymerase in a buffer with 10mM Tris-HCl (pH 8.3), 0.25 mM dNTPs (Gibco BRL, Gaithersburg, Maryland, United States), 0.2–2 mM BSA, 1.5–2.5mM MgCl_2_, 20 pmol of each primer or in total volume 25 or 50 μl containing 1–2 U of PicoMaxx High Fidelity PCR System (Taq2000 DNA polymerase, cloned Pfu DNA polymerase, and ArchaeMaxx polymerase enhancing factor in a 1× Picomaxx reaction buffer [Stratagene, La Jolla, California, United States]) and 20 pmol of each primer. The PCR conditions were 94 °C for 1–2 min, followed by 32–35 cycles of 94 °C denaturation for 30–40 s, 53–58 °C annealing for 30–40 s, and 72 °C extension for 30 s to 1 min. The PCRs were accompanied by negative controls containing the reaction solutions with no DNA (Figures 2 andS2). We found that some DNA extracts taken in high concentration may have inhibitory effects on PCR. The series of dilutions of the extracts were undertaken to identify the optimal concentration of DNA sufficient to produce PCR products after 32–35 rounds. Re-amplification by secondary PCR was not necessary. The PCR fragments were analyzed by 1.7%–2 % agarose gel stained by ethidium bromide. The PCR fragments were excised from agarose gels and purified via Qiagen columns. The PCR fragments were cloned into pGEM-T vectors (Promega, Madison, Wisconsin, United States), and the PCR fragments and clones with inserts were subjected to sequencing from mtDNA primers or vector SP6 and T4 primers using ABI PRISM BigDye kit for ABI analyzers following manufacturer's instructions (Applied Biosystems, Foster City, California, United States).

### Asian and African elephant DNA

The blood samples of theL. africana A andE. maximus A (originating from Burma) were used for DNA extraction by Qiagen (Valencia, California, United States) kits for blood DNA. We initially used long-range PCR to generate mitochondrial genome segments of ~3.0–5.0 kb. In addition, to produce longer and shorter PCR fragments, the series of primers were used in PCR conditions as described above and inTable S1. The total PCR fragments and some cloned PCR fragments were sequenced. In bothL. africana and*E. maximus,* rare mismatched sequences obviously corresponding to nuclear insertions of mitochondrial sequences were found. These diverged numts were found when some pairs of primers were used for long-range PCR using elephant DNA isolated by Qiagen kits. These pairs of primers have never been used for mammoth DNA PCR analysis. The mammoth DNA was also isolated from muscle tissue that has a relatively high proportion of mtDNA to nuclear DNA. The elephant numt sequences were removed from analysis, and the mtDNA regions were re-sequenced using other sets of primers and PCR products. Finally the complete mitochondrial genome sequences were determined from multiple independent PCR products. Comparison of the sequences determined in this study (animal A) with the corresponding*L. africana* B andE. maximus B sequences from the GenBank revealed their high similarity. The divergence of the sequences of these animals is likely due to polymorphisms. The potential sequence errors in the GenBankE. maximus mtDNA andL. africana mtDNA sequences might be of some concern. However, the results of phylogenic reconstructions remained qualitatively the same regardless of which sequences were used (ours or GenBank's) in comparative analysis with mammoth mitochondrial genome sequence.

### Sequence authentication

The work was performed in accordance with commonly accepted standards for ancient DNA analysis. Multiple measures were undertaken to exclude contamination, potential artifact DNA changes specific to post-mortem specimens, or the inclusion of numts in the mitochondrial sequence. The measures are summarized as follows. Large amounts of DNA were extracted from the well-preserved specimens. The sequencing of PCR products obtained after the first round of amplification guaranteed that no sequence errors arise due to the low number of original DNA templates and template switching during PCR. The mtDNA sequences obtained from different DNA extracts were identical. The complete mitochondrial genome sequences were determined in replication in two laboratories in different countries at different times. Apart from the VNTR region, the two mitochondrial genome sequences (MOS and UM contigs) were identical. Stringent precautions were undertaken for PCR work with ancient DNA. The work in the Moscow laboratory was conducted in a special sterile box that included two rooms designed for overnight UV irradiation. The box where the work with ancient DNA was conducted was separated from other laboratory rooms and equipment. The PCR and electrophoresis were always performed in separate rooms. No work with any animal DNA had ever been conducted in this laboratory prior to this project. The work at UMASS MS was performed in a new laboratory using novel equipment, pipettes, PCR station, and space that had never been used before for DNA analysis. No DNA work with elephants had taken place in the laboratories before the primary complete mammoth mtDNA sequence was determined. We have not found a single case of contamination by nonmammoth mtDNA (human or animal) in PCR products or clones in Moscow or in UMASS MS PCR products. Direct sequencing of PCR products from the mammoth specimen showed a high quality of chromatograms comparable to sequencing of cloned DNA (Figure S5). In all cases, the replication of sequencing was performed from different PCR products and the PCR clones. As expected, the cloned mtDNA fragments occasionally contained random mutations, transitions (A→G/T→C or C→T/G→A) corresponding to those documented previously as common type I and type II mutations in ancient DNA. The type II mutations (C→T/G→A) were predominant (>70%). These mutations were easily discriminated since they were absent in sequences of total PCR products and other clones. As a final step, the mammoth mitochondrial genome sequences were compared with GenBank sequences of other mitochondrial genomes. As we report in our phylogenetic analysis, the comparison showed the closest similarity of the mammoth sequences toL. africana andE. maximus mtDNA sequences, even when the closest relative (Sirenia) was included in the comparison. The distribution and ratio of synonymous to nonsynonymous substitutions in the mammoth sequence is similar to that found in*Elephas* and*Loxodonta* lineages and showed a high prevalence of synonymous mutations. No nucleotide mismatches were found in multiple overlapped regions determined from independent PCR fragments (refer to earlier section “Mammoth DNA”). A muscle specimen was used for DNA extraction. This type of cell tissue is particularly enriched with mitochondria, which provides a relatively high proportion of mitochondrial versus nuclear DNA.

Taken together, the methods and results indicate that contamination by exogenous DNA, numt sequences, or sequencing errors are unlikely to be a factor in our study of mammoth DNA and that the mammoth mtDNA sequence is authentic.

### Phylogenetic analysis

A multiple alignment of the seven complete mitochondrial genomes was made using the MUSCLE program [27] and checked against amino acid sequence and RNA secondary structure whenever possible. Due to an uncertain alignment in the variable region of the control region, this part of the genome (~500 bp) was excluded from all analyses. The resulting alignment (referred to as the complete mitochondrial alignment throughout the text) was used for all of our phylogenetic and comparative analyses. To estimate synonymous and nonsynonymous nucleotide divergence, a concatenated alignment of all protein-coding genes was used, and dn and ds values were estimated with the Pamilo-Bianchi-Li method as implemented in MEGA [28]. All analyses performed with the complete mitochondrial alignment were repeated using the concatenated alignment of all protein and RNA genes (discarding ~1,000 bp of nontranscribed sequence), and all of the results remained qualitatively unchanged.

To reconstruct the tree topology, parsimony and neighbor-joining methods were used as implemented in MEGA [28] with default parameters and 10,000 bootstrap replicates [28–31]. Bayesian inference of phylogeny was done with MrBayes [29] with two different prior models: the General Time Reversible model [28,29] and the HKY model [28,29], which estimates fewer rate parameters. Both models were run assuming a gamma-distribution of substitution rates across sites for 1 million iterations (mcmc ngen = 1000000 in MrBayes). Phylogenetic trees using individual gene sequences were tested with Bayesian inference set to the HKY model (Table S3). An ML ratio test was performed for ML values obtained for the possible topologies of the three Elephantinae species. The topology withM. primigenius andE. maximus as sister species had a log ML of −42440.629, compared with −42465.549 and −42456.866 for the topology linkingE. maximus withL. africana andM. primigenius with*L. africana,* respectively (*p* < 0.01; ML ratio test). The log ML scores reported here are averages of log ML scores obtained for 10,000,000 iterations of the BEAST [30] program and ran under the HKY [28,29] model with set tree topologies.

The molecular clock was tested with the likelihood ratio test, using the log likelihood scores obtained with the baseml program in PAML [32] when the dataset was analyzed with the assumption of no molecular clock (clock = 0 in baseml) and a local clock that tested the difference between the species in question (clock = 2 in baseml). To estimate the branch length for synonymous and nonsynonymous substitutions, the branches of interest were assumed to have different dn/ds ratios (model = 2 in codeml) and an absence of a molecular clock (clock = 0 in codeml). To estimate the divergence time of*M. primigenius,* a heuristic rate smoothing procedure for ML estimates [16] was used as implemented in PAML [32]. The number of synonymous and nonsynonymous substitutions (Table S4) was obtained by parsimony using the African elephant as the outgroup for the mammoth–Asian elephant comparison and dugong as the outgroup for the mammoth–African elephant and Asian–African elephant comparisons without correcting for multiple substitutions and with the assumption that the outgroup accurately resembles the ancestral state. These results yielded were quantitatively very similar to those obtained with a ML approach that corrected for multiple substitutions (unpublished). Control files for all stand-alone programs ran here and other methodological materials are available on request.

## Supporting Information

Figure S1 Mammoth Specimen Found in Siberia (A) Restored exhibit of mammoth Enmyn leg found in the Enmynveem River valley, Chukotka, northeastern Siberia. (B) The geographic location of the find is designated by the red dot on the map.
(2.3 MB PDF).

Figure S2 Electrophoresis Analysis of the PCR Products Used for Sequencing ofM. primigenius Mitochondrial Genome (“MOS Mammoth mtDNA”) A and B agarose gel panels.
The negative controls are shown in (A). * designates the area of oligonucleotide primer dimers formed in PCR.
(631 KB PDF).

Figure S3 Electrophoresis Analysis of the PCR Products Used for Sequencing ofM. primigenius Mitochondrial Genome (“UM Mammoth mtDNA”) A–D agarose gel panels. * designates lines with low amount of PCR products.
(522 KB PDF).

Figure S4 Extreme Heteroplasmy in Hypervariable Region HVR (VNTR Region) in the Control Region of Mitochondrial Genome ofM. primigenius
(A) Electrophoresis of PCR products from two DNA extracts (m1 and m22) and (B) corresponding PCR clones with VNTR region mtDNA insertions showed variability in their length. (C) Nucleotide sequences of cloned PCR products with VNTR. The sequence for the longest VNTR was incorporated in a reported complete sequence of*M. primigenius.*
(455 KB PDF).

Figure S5 Sequencing Electrophoresis Data for the Mitochondrial Genome ofM. primigenius with High-Quality Chromatograms of Direct Sequencing of PCR Products, in Comparison with the Cloned PCR Fragments Three mtDNA regions are shown: (A) L11-H11, (B) L17-H17, and (C) L22-H22. Lines 1 and 2 (A) and line 1 (B and C) from the bottom are the PCR products. Other lines are cloned sequences determined from opposite strands. The rare mutations are observed in cloned sequences but not in direct PCR product sequences.
(503 KB PDF).

Figure S6 Comparison ofM. primigenius mtDNA Sequences in Individual Clones and Direct PCR Products The rare, random “ancient DNA” mutations were identified in cloned sequences. No sequences with identical series of mutations were found in individual clones, providing evidence that the PCR products were obtained from the significant amount of original DNA templates. Mutations were confirmed by sequencing of both DNA strands. The G→A and C→T mutations (type II mutations) were prevalent, as is expected for ancient “DNA” modifications.
(22 KB PDF).

Table S1 Primer Oligonucleotides and Predicted PCR Fragments Used for Sequencing of Complete Mitochondrial Genome of*M. primigenius, E. maximus,* and*L. africana*
Redundant primer oligonucleotides were used to amplify mammoth DNA. The sequences of mitochondrial genomes ofE. maximus andL. africana were determined using both long-range PCR fragments and short PCR fragments.
(30 KB DOC).

Table S2 Different Types of Substitutions Revealed by Pairwise Comparisons of Elephantinae Genomes Number of synonymous and nonsynonymous substitutions calculated with the modified Nei-Gojobory method as implemented in MEGA [28]; the results were independent of the transition/transversion ratio.
(25 KB DOC).

Table S3 Bayesian Phylogeny Inference [29] of Elephantinae Using Single Mitochondrial Protein Coding and rRNA Genes Posterior probabilities are shown in parentheses.
(26 KB DOC).

Table S4 Lineage-Specific Substitutions Inferred by Pairwise Comparisons of Elephantinae Genomes and Polarized with One Outgroup We used the sequences ofE. maximus andL. africana determined in this study for these comparisons. Pairwise comparisons of Elephantinae species using a single outgroup to polarize substitutions suggested that theM. primigenius–E. maximus clade evolves faster than theL. africana lineage in both synonymous and nonsynonymous sites.
(26 KB DOC).

Table S5 Nucleotide Diversity Inferred by Pairwise Comparisons of DifferentM. primigenius mtDNA Sequences Sequences are reported in the following studies: DQ316067 (present study), NC_007596 [20], D83047 [18], and D50841–D50842 [19].
(31 KB DOC).

Table S6 Genome-Specific Nonsynonymous and Synonymous Substitutions Inferred by Pairwise Comparisons of Protein-Coding Genes from TwoM. primigenius Genomes (DQ316067 versus NC_007596) and Polarized withE. maximus (DQ316068) Sequence A large number of nonsynonymous substitutions was observed in the*ND2* gene (seven nonsynonymous versus two synonymous mutations in NC_007596 accumulated in a short ~360-bp region; whereas the DQ316067 sequence determined in this study had zero nonsynonymous and one synonymous substitution in the same gene).
(38 KB DOC).

Table S7 Genome-Specific Substitutions Inferred by Pairwise Comparison of TwoM. primigenius Genomes (DQ316067 versus NC_007596) and Polarized withE. maximus (DQ316068) Sequence While the C→T and G→A substitutions may be a sign of ancient DNA modification (most frequent type II mutations), they represent also real polymorphisms. The NC_007596 mammoth genome shows more of these substitutions, suggesting that a few of these substitutions may have occurred after death of the sampled mammoth; however, this difference is not statistically significant (Fisher's exact test*p* > 0.05).
(25 KB DOC).

### Accession numbers

The GenBank (http://www.ncbi.nlm.nih.gov) accession numbers for the complete mitochondrial genome sequences determined in this paper areE. maximus (DQ316068),L. africana (DQ316069), andM. primigenius (DQ316067). The GenBank accession numbers for otherM. primigenius mtDNA sequences are D83047 [18], D50841–D50842 [19], and NC_007596 [20]. The GenBank accession numbers for mitochondrial genomes ofE. maximus andL. africana [33] are NC_005129 and NC_000934, respectively, and forD. dugon andP. capensis are NC_003314 and NC_004919, respectively.

## Abbreviations

bp: base pair
ML: maximum likelihood
mtDNA: mitochondrial DNA
numt: nuclear mitochondrial DNA
UMASS MS: University of Massachusetts Medical School
VNTR: variable number of tandem repeats
