# Supplementary material for: Complete Mitochondrial Genome and Phylogeny of Pleistocene MammothMammuthus primigenius
Source: PLoS Biol. 2006 Feb 7;4(3):e73. doi: 10.1371/journal.pbio.0040073 (PMC1360101; doi:10.1371/journal.pbio.0040073)
Supplement: Supplementary file 1 [file pbio.0040073.sg001.pdf]

Figure S1

A

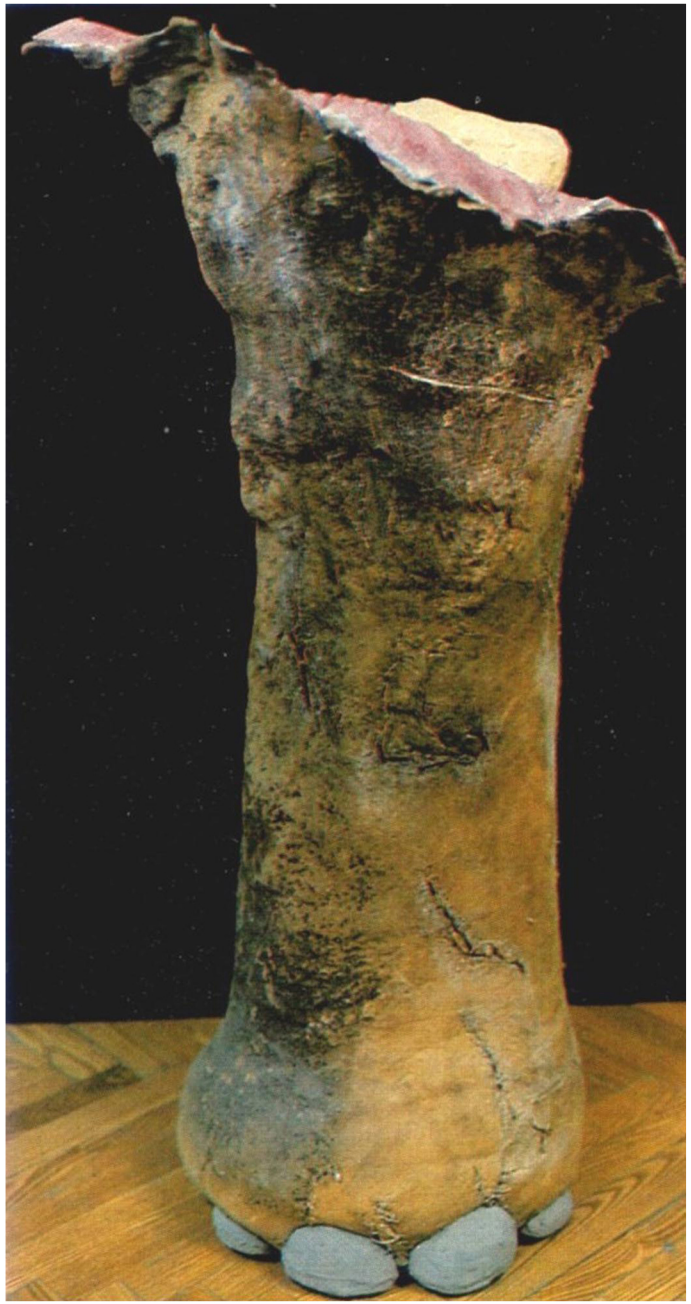

B

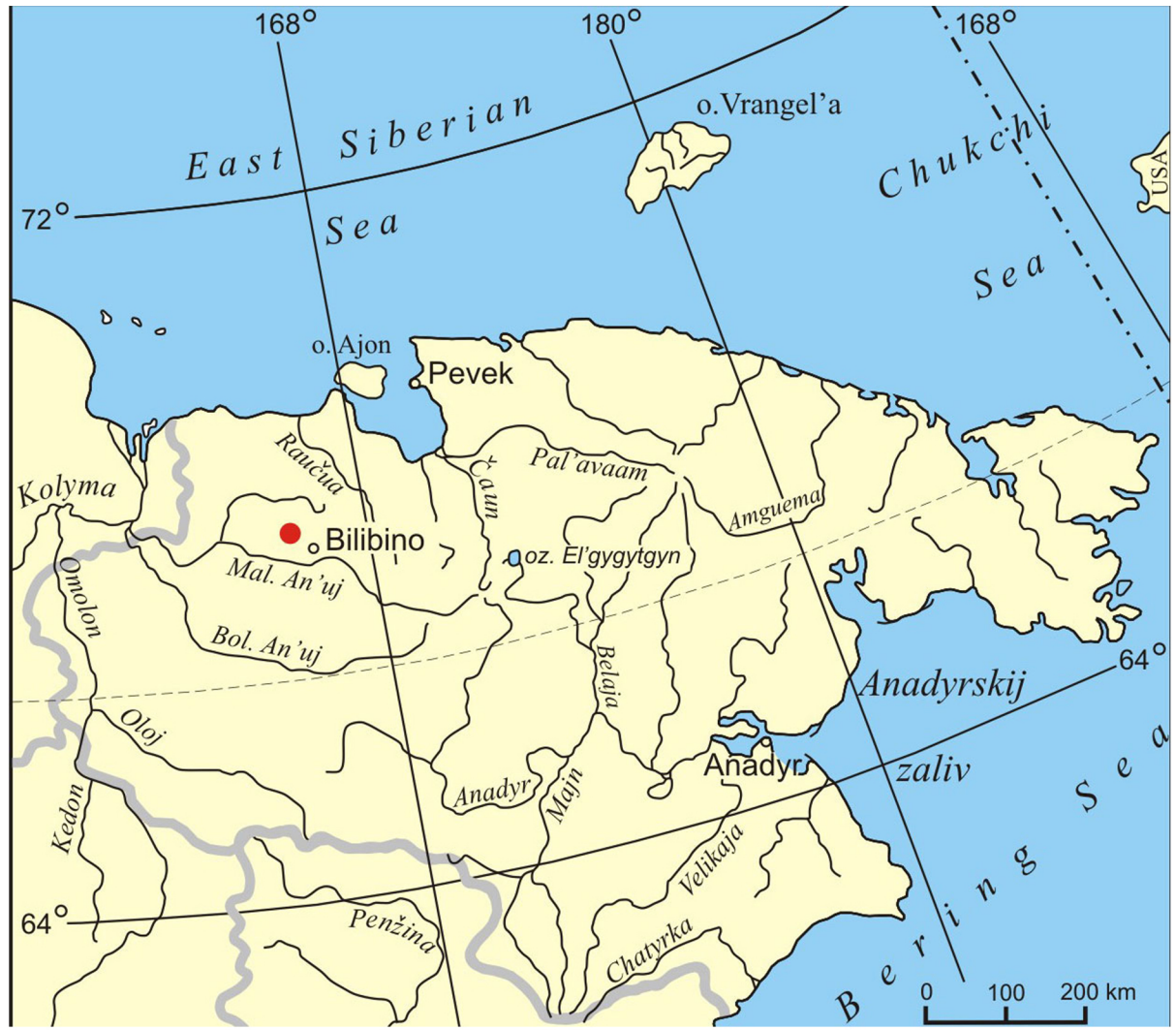
168°
180°
168°
o.Vrangel'a
East Siberian Sea
Chukchi Sea
USA
72°
o. Ajon
Pevek
Raučua
Čaun
Pal'avaam
Amguema
Kolyma
Bilibino
oz. El'gygytgyn
Mal. An'uj
Omolon
Bol. An'uj
Belaja
64°
Anadyrskij zaliv
Oloj
Anadyr
Anadyr
Majn
Kedon
Velikaja
Bering Sea
64°
Penžina
Chatyrka
0 100 200 km
