# Supplementary material for: Complete Mitochondrial Genome and Phylogeny of Pleistocene MammothMammuthus primigenius
Source: PLoS Biol. 2006 Feb 7;4(3):e73. doi: 10.1371/journal.pbio.0040073 (PMC1360101; doi:10.1371/journal.pbio.0040073)
Supplement: Supplementary file 2 [file pbio.0040073.sg002.pdf]

Figure S2

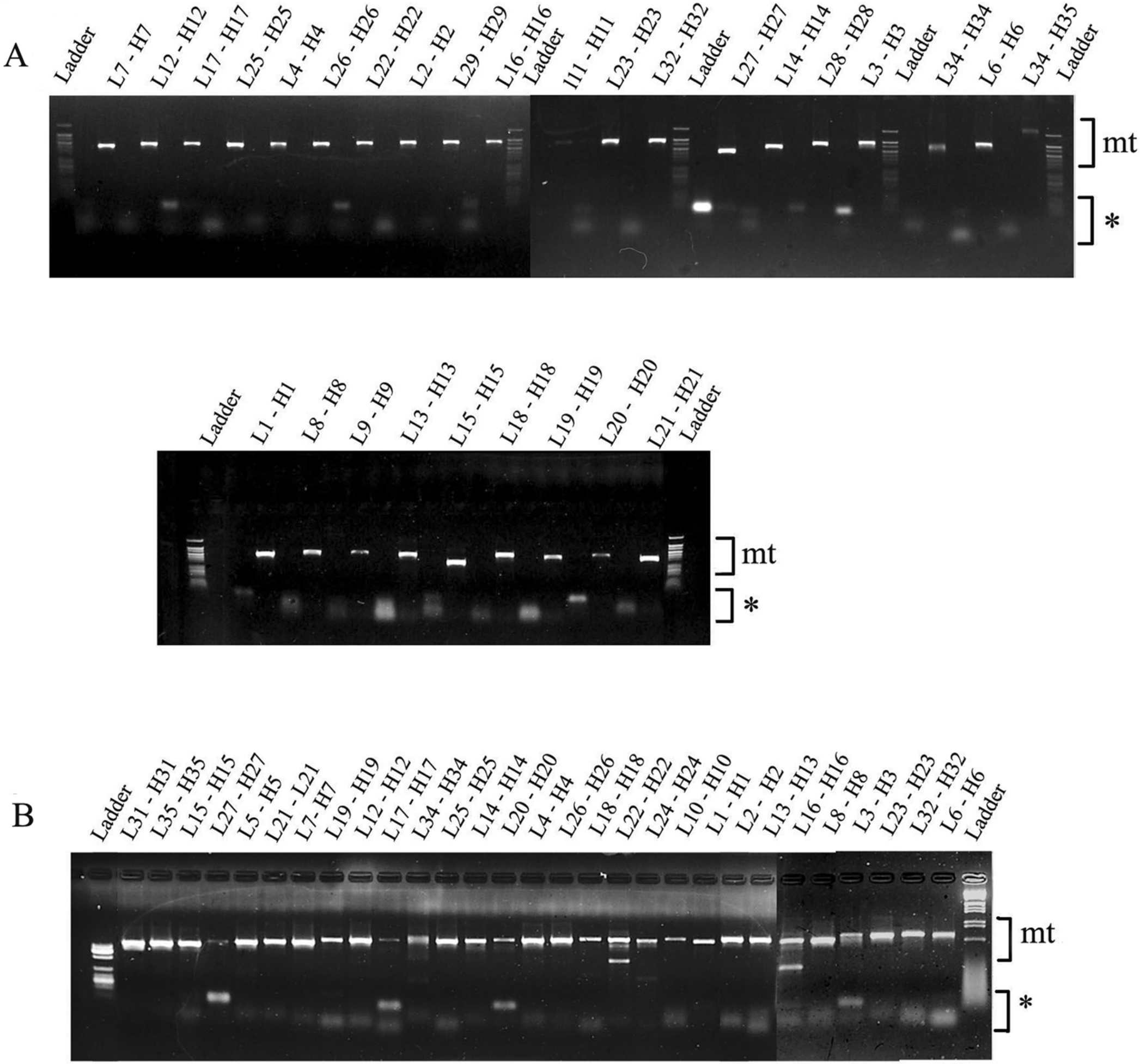
A
Ladder
L7 - H7
L12 - H12
L17 - H17
L25 - H25
L4 - H4
L26 - H26
L22 - H22
L2 - H2
L29 - H29
L16 - H16
Ladder
l11 - H11
L23 - H23
L32 - H32
Ladder
L27 - H27
L14 - H14
L28 - H28
L3 - H3
Ladder
L34 - H34
L6 - H6
L34 - H35
Ladder
mt
*
Ladder
L1 - H1
L8 - H8
L9 - H9
L13 - H13
L15 - H15
L18 - H18
L19 - H19
L20 - H20
L21 - H21
Ladder
mt
*
B
Ladder
L31 - H31
L35 - H35
L15 - H15
L27 - H27
L5 - H5
L21 - L21
L7 - H7
L19 - H19
L12 - H12
L17 - H17
L34 - H34
L25 - H25
L14 - H14
L20 - H20
L4 - H4
L26 - H26
L18 - H18
L22 - H22
L24 - H24
L10 - H10
L1 - H1
L2 - H2
L13 - H13
L16 - H16
L8 - H8
L3 - H3
L23 - H23
L32 - H32
L6 - H6
Ladder
mt
*
