# Supplementary material for: Complete Mitochondrial Genome and Phylogeny of Pleistocene MammothMammuthus primigenius
Source: PLoS Biol. 2006 Feb 7;4(3):e73. doi: 10.1371/journal.pbio.0040073 (PMC1360101; doi:10.1371/journal.pbio.0040073)
Supplement: Supplementary file 3 [file pbio.0040073.sg003.pdf]

Figure S3

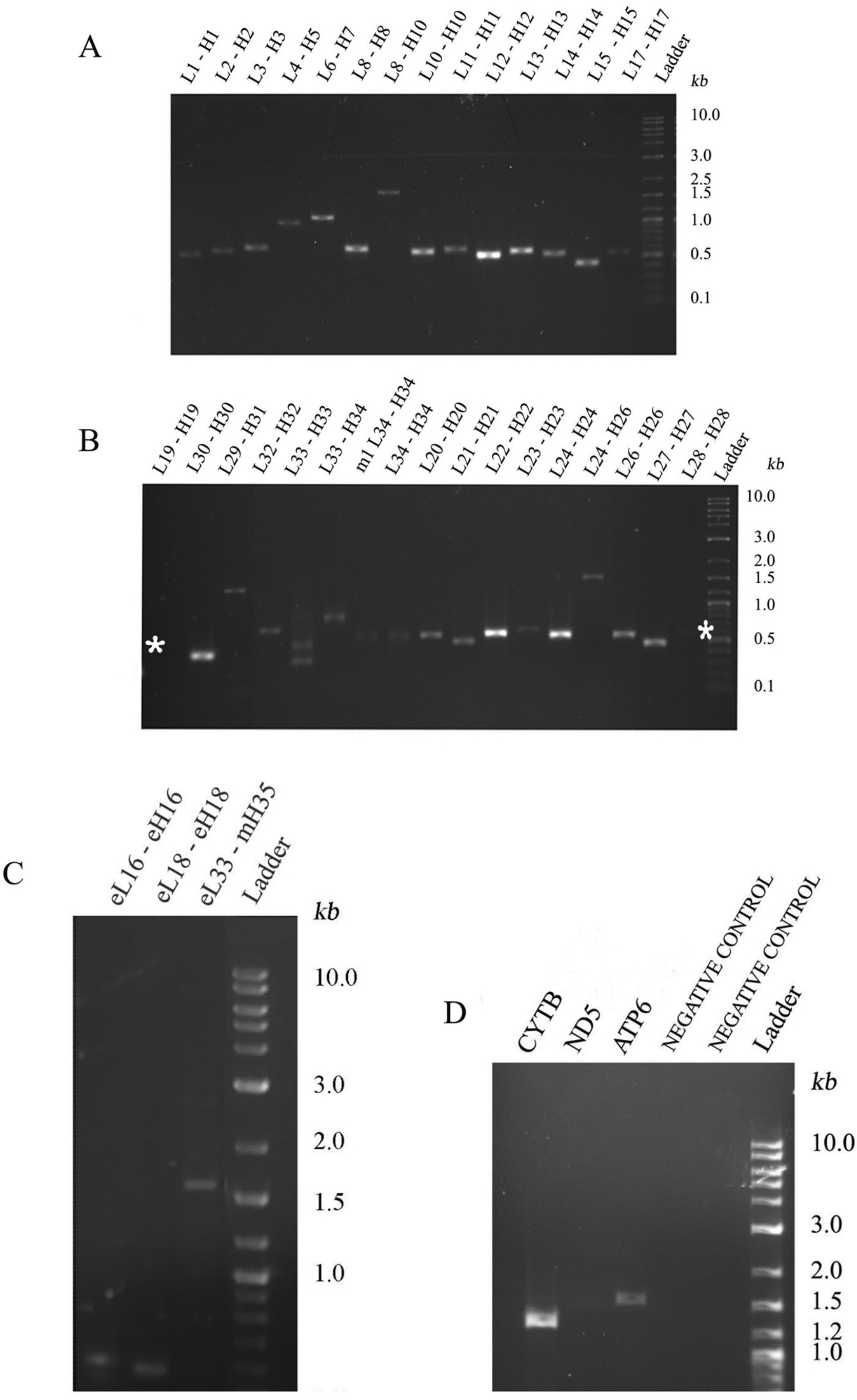
A
L1 - H1
L2 - H2
L3 - H3
L4 - H5
L6 - H7
L8 - H8
L8 - H10
L10 - H10
L11 - H11
L12 - H12
L13 - H13
L14 - H14
L15 - H15
L17 - H17
Ladder
kb
10.0
3.0
2.5
1.5
1.0
0.5
0.1
B
L19 - H19
L30 - H30
L29 - H31
L32 - H32
L33 - H33
L33 - H34
m1 L34 - H34
L34 - H34
L20 - H20
L21 - H21
L22 - H22
L23 - H23
L24 - H24
L24 - H26
L26 - H26
L27 - H27
L28 - H28
Ladder
kb
10.0
3.0
2.0
1.5
1.0
0.5
0.1
*
*
C
eL16 - eH16
eL18 - eH18
eL33 - mH35
Ladder
kb
10.0
3.0
2.0
1.5
1.0
D
CYTB
ND5
ATP6
NEGATIVE CONTROL
NEGATIVE CONTROL
Ladder
kb
10.0
3.0
2.0
1.5
1.2
1.0
