# Supplementary material for: Complete Mitochondrial Genome and Phylogeny of Pleistocene MammothMammuthus primigenius
Source: PLoS Biol. 2006 Feb 7;4(3):e73. doi: 10.1371/journal.pbio.0040073 (PMC1360101; doi:10.1371/journal.pbio.0040073)
Supplement: Supplementary file 4 [file pbio.0040073.sg004.pdf]

Figure S4

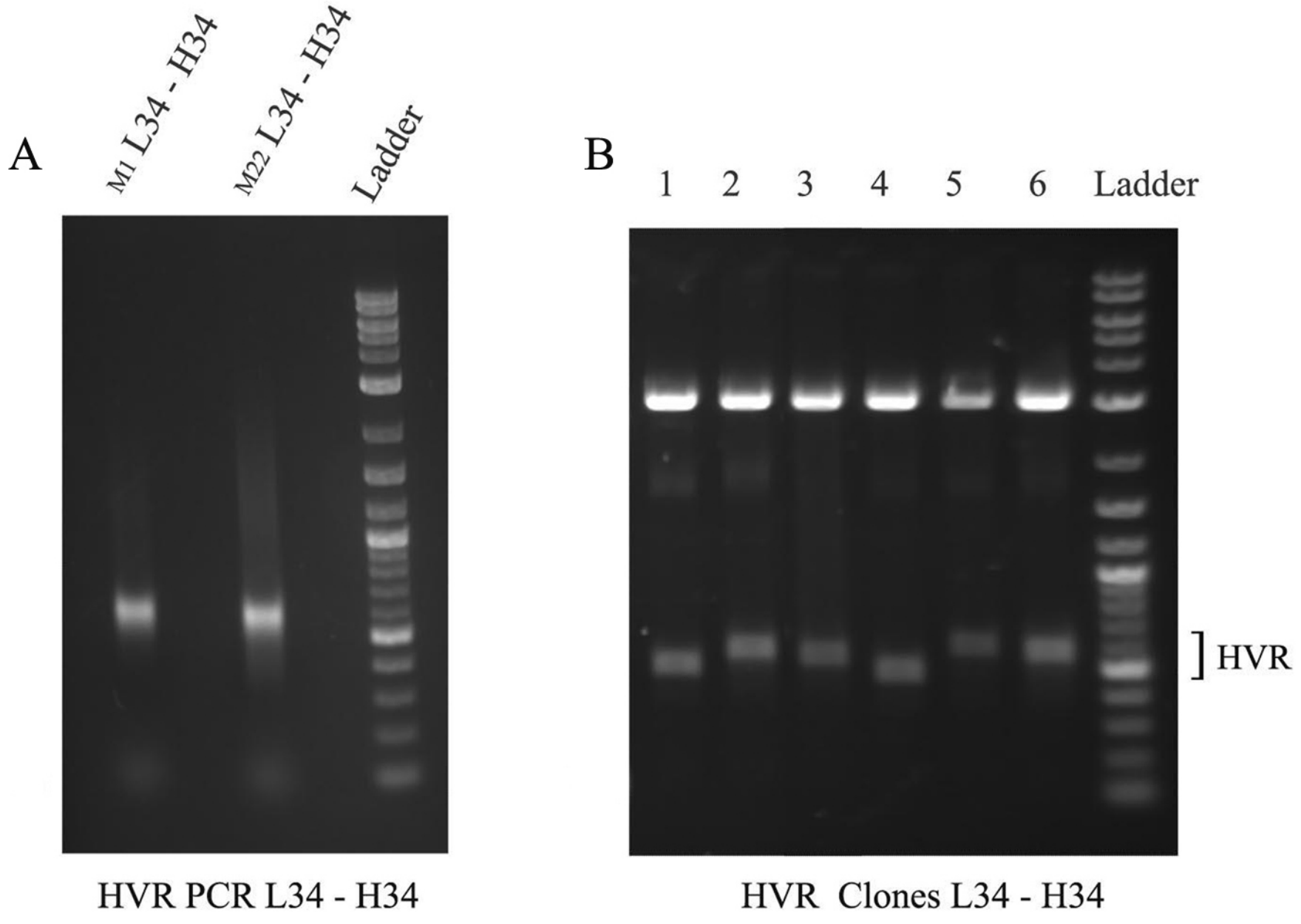
A
M1 L34 - H34
M22 L34 - H34
Ladder
B
1
2
3
4
5
6
Ladder
HVR
HVR PCR L34 - H34
HVR Clones L34 - H34

Figure S4 C, page 1

```
                           *         *         *         *         50
MAM22_34_3  :     ATTTTTTAAACCTAAATTCCCAGCGTACACGCATACGCACATGCATACGT
MAM22_34_4  :     ATTTTTTAAACCTAAATTCCCAGCGTACACGCATACGCACATGCATACGT
MAM22_34_7  :     ATTTTTTAAACCTAAATTCCCAGCGTACACGCATACGCACATGCATACGT
MAM22_34_8  :     ATTTTTTAAACCTAAATTCCCAGCGTACACGCATACGCACATGCATACGT
MAM22_34_9  :     ATTTTTTAAAC-TAAATTCCCAGCGTACACGCATACGCACATGCATACGT

                           *         *         *         *         100
MAM22_34_3  :     AC----------ACGTACACGTACGTACACGTACGTACACGTACGTACAC
MAM22_34_4  :     AC----------ACGTACACGTACGTACACGTACGTACACGTACGTACAC
MAM22_34_7  :     ACACGTACATGTACGTACACGTACGTACACGTACGTACACGTACGTACAC
MAM22_34_8  :     AC----------ACGTACACGTACGTACACGTACGTACACGTACGTACAC
MAM22_34_9  :     AC----------ACGTACACGTACGTACACGTACGTACACGTACGTACAC

                           *         *         *         *         150
MAM22_34_3  :     GCACGTACACGTACACGTACACGCACGTACACGTACACGTACACGTACGT
MAM22_34_4  :     GCATGTACACGTACACGTACACGCACGTACACGTACACGTATACGTACGT
MAM22_34_7  :     GCACGTACACGTACACGTACACGCACGTACACGTACACGTACACGTACGT
MAM22_34_8  :     GCACGTACACGTACACGTACACGCA-------------------------
MAM22_34_9  :     GCACGTACACGTACACGTAC------------------------------

                           *         *         *         *         200
MAM22_34_3  :     ACACGCACGTACACGTACACGTACACGCATACGCATACGCATACGCATAC
MAM22_34_4  :     ACACGCACGTACACGTACACGTACACGCATACGCATACGCATACGCATAC
MAM22_34_7  :     ACACGCACGTACACGTACACGTAC--------------------------
MAM22_34_8  :     -----------------------------------------TACGCATAC
MAM22_34_9  :     --------------------------------------------------

                           *         *         *         *         250
MAM22_34_3  :     GCATACGCATACGCATACGCATACGCATACGCATACGCATACGCATACGC
MAM22_34_4  :     GCATACGCATACGCATACGCATACGCATACGCATACGCATACGCATACGC
MAM22_34_7  :     ----------------------------------------------ACGC
MAM22_34_8  :     GCATACGCATACGCATACGCATACGCATACGCATACGCATACGCATACGC
MAM22_34_9  :     ----ACGCATACGCATACGCATACGCATACGCATACGCATACGCATACGC

                           *         *         *         *         300
MAM22_34_3  :     ATACGCATACGCATACGCATACGCATACGCATACGCATACGCATACGCAT
MAM22_34_4  :     ATACGCATACGCATACGCATACGCATACGCATACGCATACGCATACGCAT
MAM22_34_7  :     ATACGCATACGCATACGCATACGCATACGCATACGCATACGCATACGCAT
MAM22_34_8  :     ATACGCATACGCATACGCATACGCATACGCATACGCATACGCATACGCAT
MAM22_34_9  :     ATACGCATACGCATACGCATACGCATACGCATACGCATACGCATACGCAT

                           *         *         *         *         350
MAM22_34_3  :     ACGCATACGTATACGTATACG-----------------------------
MAM22_34_4  :     ACGCATACGCATACGCATACGCATACGCATACGCATACGCATACGCATAC
MAM22_34_7  :     ACGCATACGCATACGCATACGCATACGCATACGCATACGCATACGCATAC
MAM22_34_8  :     ACGTATACGCATACGCATACGCATACGCATACGCATACGCATACGCATAC
MAM22_34_9  :     ACGCATACGCATACGCATACGCATACGCATACGCATACGCATACGCATAC
```

Figure S4 C, page 2

```
                          *         *         *         *         400
MAM22_34_3  :    --------------------------------------------------
MAM22_34_4  :    GCATACGCATACGCATACGCATACGCATACG-------------------
MAM22_34_7  :    GCATACGCATACGCATACGCATACGCATACG-------------------
MAM22_34_8  :    GCATACGCATACGCATACGCATACGCATACGCATACGCATACGCATACGC
MAM22_34_9  :    GCATACGCATACGCATACGCATACGCATACGCATACGCATACGCATACGC

                          *         *         *         *         450

MAM22_34_3  :    --------------------------------------------------
MAM22_34_4  :    --------------------------------------------------
MAM22_34_7  :    --------------------------------------------------
MAM22_34_8  :    ATACGCATACGCATACGCATACGCATACGCATACGCATACGCATACGCAT
MAM22_34_9  :    ATACGCATACGCATACGCATACGCATACGCATACGCATACGCATACGCAT

                          *         *         *         *         500
MAM22_34_3  :    ---------------------------------------TAACCACTATG
MAM22_34_4  :    ---------------------------TATACGTATACGTAACCACTATG
MAM22_34_7  :    --------------------------------------------------
MAM22_34_8  :    ACGCATACGCATACGCATACGCATACGTATACGTATACGTAACCACTATG
MAM22_34_9  :    ACGCATACGCATACGCATACGCATACGTATACGTATACGTAAN-ACTATG

                          *         *         530
MAM22_34_3  :    TAACTATCTCTTCAAACCCCCCTTACCC
MAM22_34_4  :    TAACTATCTCTTCAAACCCCCCTTACCC
MAM22_34_7  :    ----------------------------
MAM22_34_8  :    TAACTATCTCTTCAAACCCCCCTTACTC
MAM22_34_9  :    TAACTATCTCTTCAAACCCCCCTTACCC
```
