# Supplementary material for: Complete Mitochondrial Genome and Phylogeny of Pleistocene MammothMammuthus primigenius
Source: PLoS Biol. 2006 Feb 7;4(3):e73. doi: 10.1371/journal.pbio.0040073 (PMC1360101; doi:10.1371/journal.pbio.0040073)
Supplement: Supplementary file 5 [file pbio.0040073.sg005.pdf]

Figure S5 A

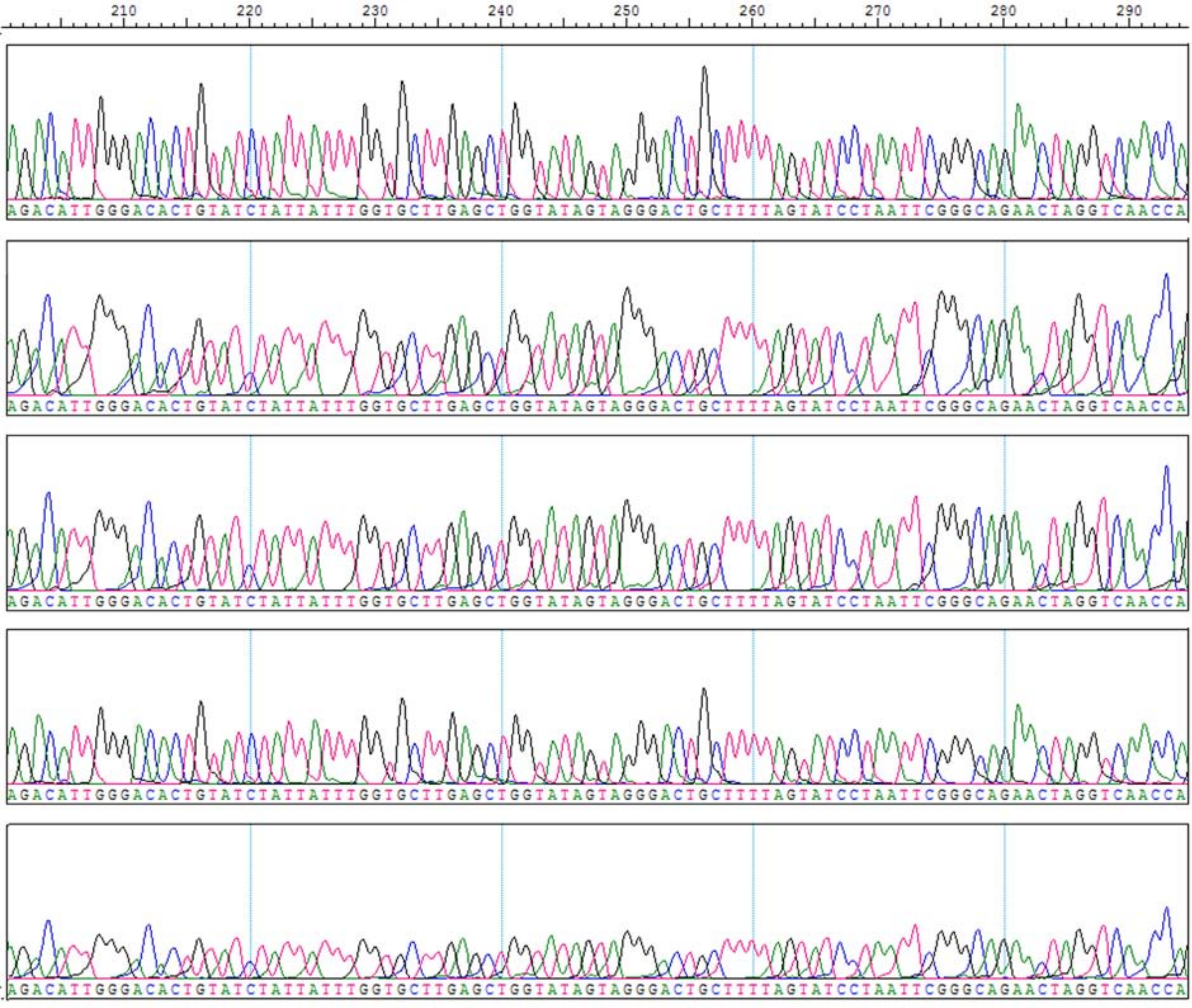
210
220
230
240
250
260
270
280
290
AGACATTGGGACACTGTATCTATTATTTGGTGCTTGAGCTGGTATAGTAGGGACTGCTTTTAGTATCCTAATTCGGGCAGAACTAGGTCAACCA
AGACATTGGGACACTGTATCTATTATTTGGTGCTTGAGCTGGTATAGTAGGGACTGCTTTTAGTATCCTAATTCGGGCAGAACTAGGTCAACCA
AGACATTGGGACACTGTATCTATTATTTGGTGCTTGAGCTGGTATAGTAGGGACTGCTTTTAGTATCCTAATTCGGGCAGAACTAGGTCAACCA
AGACATTGGGACACTGTATCTATTATTTGGTGCTTGAGCTGGTATAGTAGGGACTGCTTTTAGTATCCTAATTCGGGCAGAACTAGGTCAACCA
AGACATTGGGACACTGTATCTATTATTTGGTGCTTGAGCTGGTATAGTAGGGACTGCTTTTAGTATCCTAATTCGGGCAGAACTAGGTCAACCA

Figure S5 B

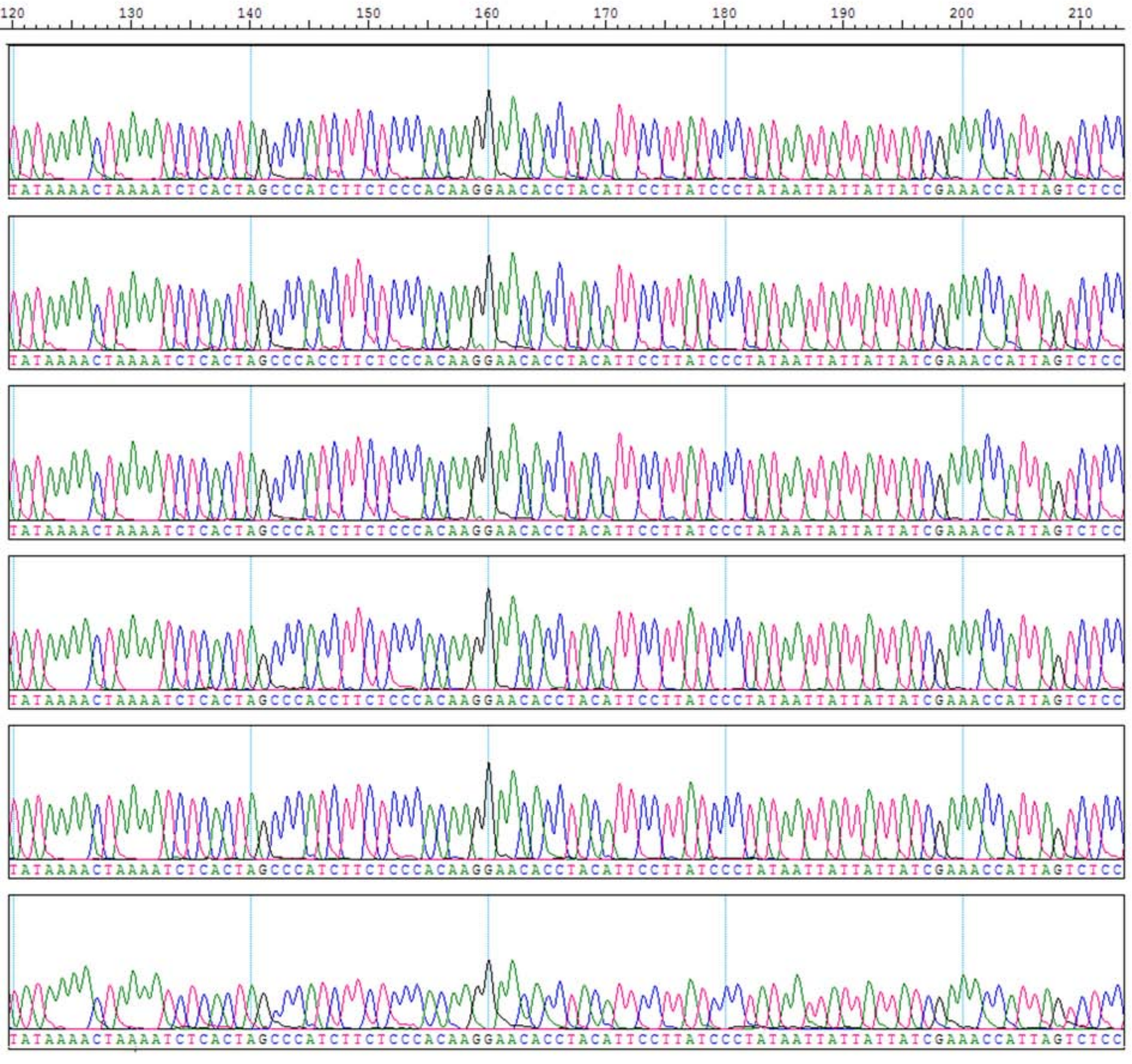
120 130 140 150 160 170 180 190 200 210
TATAAAACTAAAATCTCACTAGCCCATCTTCTCCCACAAGGAACACCTACATTCCTTATCCCTATAATTATTATTATCGAAACCATTAGTCTCC
TATAAAACTAAAATCTCACTAGCCCACCTTCTCCCACAAGGAACACCTACATTCCTTATCCCTATAATTATTATTATCGAAACCATTAGTCTCC
TATAAAACTAAAATCTCACTAGCCCATCTTCTCCCACAAGGAACACCTACATTCCTTATCCCTATAATTATTATTATCGAAACCATTAGTCTCC
TATAAAACTAAAATCTCACTAGCCCACCTTCTCCCACAAGGAACACCTACATTCCTTATCCCTATAATTATTATTATCGAAACCATTAGTCTCC
TATAAAACTAAAATCTCACTAGCCCATCTTCTCCCACAAGGAACACCTACATTCCTTATCCCTATAATTATTATTATCGAAACCATTAGTCTCC
TATAAAACTAAAATCTCACTAGCCCATCTTCTCCCACAAGGAACACCTACATTCCTTATCCCTATAATTATTATTATCGAAACCATTAGTCTCC

Figure S5 C

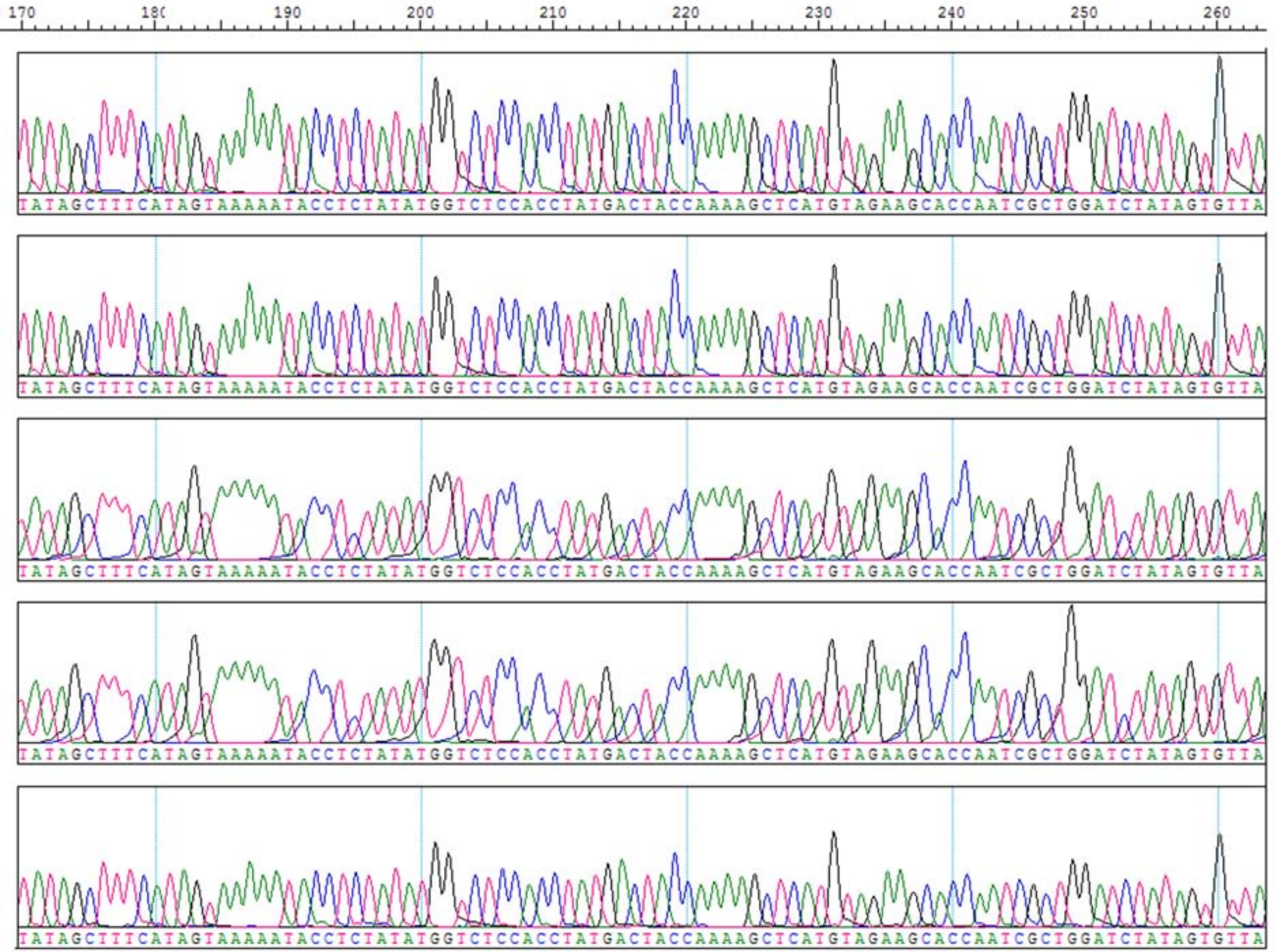
170 180 190 200 210 220 230 240 250 260
TATAGCTTTCATAGTAAAAATACCTCTATATGGTCTCCACCTATGACTACCAAAAGCTCATGTAGAAGCACCAATCGCTGGATCTATAGTGTTA
TATAGCTTTCATAGTAAAAATACCTCTATATGGTCTCCACCTATGACTACCAAAAGCTCATGTAGAAGCACCAATCGCTGGATCTATAGTGTTA
TATAGCTTTCATAGTAAAAATACCTCTATATGGTCTCCACCTATGACTACCAAAAGCTCATGTAGAAGCACCAATCGCTGGATCTATAGTGTTA
TATAGCTTTCATAGTAAAAATACCTCTATATGGTCTCCACCTATGACTACCAAAAGCTCATGTAGAAGCACCAATCGCTGGATCTATAGTGTTA
TATAGCTTTCATAGTAAAAATACCTCTATATGGTCTCCACCTATGACTACCAAAAGCTCATGTAGAAGCACCAATCGCTGGATCTATAGTGTTA
