# Supplementary material for: Complete Mitochondrial Genome and Phylogeny of Pleistocene MammothMammuthus primigenius
Source: PLoS Biol. 2006 Feb 7;4(3):e73. doi: 10.1371/journal.pbio.0040073 (PMC1360101; doi:10.1371/journal.pbio.0040073)
Supplement: Supplementary file 6 [file pbio.0040073.sg006.pdf]

Figure S6 A, page 1

```
                    *        20         *        40         *
fragm 11 : CGCCTTGAAGGAGGGGGAAAAAGGCGGGAGAAGCCCCGGCAGAATTGAAGCTGCTT :  56
MAM 11 1 : ........................................................ :  56
MAM 11 4 : T....................................................... :  56
MAM 11 2 : ..T..................................................... :  56
MAM 11 6 : ..TT.................................................... :  56
MAM 11 3 : ........................T............................... :  56
MAM_11_5 : .........................A.............................. :  56

             60         *        80         *       100         *
fragm 11 : TTTTCGAATTTGCAGTTCGACATGTTTACACTTTCAAGGCCTGGTAAAAAGAGATT : 112
MAM 11 1 : ........................................................ : 112
MAM 11 4 : ........................................................ : 112
MAM 11 2 : ........................................................ : 112
MAM 11 6 : ........................................................ : 112
MAM 11 3 : ........................................................ : 112
MAM_11_5 : ........................................................ : 112

                120         *       140         *       160
fragm 11 : ACTCTCTGTGGTTAGATTTACAGTCTAATACTTACTCAGCCATTTTACCTATGTTT : 168
MAM 11 1 : ........................................................ : 168
MAM 11 4 : ........................................................ : 168
MAM 11 2 : ........................................................ : 168
MAM 11 6 : ........................................................ : 168
MAM 11 3 : ........................................................ : 168
MAM_11_5 : ........................................................ : 168

            *       180         *       200         *       220
fragm 11 : GCTAACCGCTGACTATATTCAACAAACCACAAAGACATTGGGACACTGTATCTATT : 224
MAM 11 1 : ........................................................ : 224
MAM 11 4 : ........................................................ : 224
MAM 11 2 : ........................................................ : 224
MAM 11 6 : ........................................................ : 224
MAM 11 3 : ........................................................ : 224
MAM_11_5 : ........................................................ : 224

                *       240         *       260         *       280
fragm 11 : ATTTGGTGCTTGAGCTGGTATAGTAGGGACTGCTTTTAGTATCCTAATTCGGGCAG : 280
MAM 11 1 : ........................................................ : 280
MAM 11 4 : ........................................................ : 280
MAM 11 2 : ........................................................ : 280
MAM 11 6 : ........................................................ : 280
MAM 11 3 : ........................................................ : 280
MAM 11 5 : ........................................................ : 280
                    *       300         *       320         *
fragm 11 : AACTAGGTCAACCAGGCTCTCTTCTTGGAGACGACCAAATCTATAATGTTATTGTC : 336
MAM 11 1 : ........................................................ : 336
MAM 11 4 : ........................................................ : 336
MAM 11 2 : ........................................................ : 336
MAM 11 6 : ........................................................ : 336
MAM 11 3 : ........................................................ : 336
MAM_11_5 : ........................................................ : 336
```

Figure S6 A, page 2

```
            340         *       360         *       380         *
fragm 11 : ACAGCACACGCCTTTGTAATAATCTTCTTTATAGTTATGCCAATTATAATTGGAGG : 392
MAM 11 1 : ........................................................ : 392
MAM 11 4 : .T...................................................... : 392
MAM 11 2 : ........................................................ : 392
MAM 11 6 : ......................................................A. : 392
MAM 11 3 : ........................................................ : 392
MAM_11_5 : ........................................................ : 392

                400         *       420         *       440
fragm 11 : CTTTGGAAACTGATTAATTCCACTTATAATCGGAGCACCTGATATAGCTTTTCCTC : 448
MAM 11 1 : ........................................................ : 448
MAM 11 4 : ........................................................ : 448
MAM 11 2 : ........................................................ : 448
MAM 11 6 : ........................................................ : 448
MAM 11 3 : ........................................................ : 448
MAM_11_5 : ........................................................ : 448

            *       460         *       480         *       500
fragm 11 : GAATAAACAATATGAGTTTTTGACTACTACCTCCATCTTTCCTACTACTCTTGGCA : 504
MAM 11 1 : ........................................................ : 504
MAM 11 4 : ........................................................ : 504
MAM 11 2 : ................C....................................... : 504
MAM 11 6 : ........................................................ : 504
MAM 11 3 : ........................................................ : 504
MAM_11_5 : ........................................................ : 504

                *       520         *       540         *       560
fragm 11 : TCTTCCATAGTAGAAGCTGGGACAGGCACTGGTTGGACCGTATACCCTCCCCTGGC : 560
MAM 11 1 : ............A...............................T........... : 560
MAM 11 4 : ........................................................ : 560
MAM 11 2 : ........................................................ : 560
MAM 11 6 : ........................................................ : 560
MAM 11 3 : ........................................................ : 560
MAM_11_5 : ........................................................ : 560

                    *
fragm 11 : AGGAAACCTAGCCCATGCA : 579
MAM 11 1 : ................... : 579
MAM 11 4 : ................... : 579
MAM 11 2 : ................... : 579
MAM 11 6 : ................... : 579
MAM 11 3 : ................... : 579
MAM_11_5 : ................... : 579
```

Figure S6 B

```
                      *        20         *        40         *
fragm17    : TCTATTACCCTATTCATTCGCTCCTACAGCACAACTAACCGTAAACTTAAGCAT :  54
MAM22 17 2 : ...................................................... :  54
MAM22 17 3 : ...................................................... :  54
MAM22_17_5 : ...................................................... :  54

                 60         *        80         *       100
fragm17    : AGCAATCCCCCTATGGACTGGTACAGtTATCCTGGGCTTCCGATATAAAACTAA : 108
MAM22 17 2 : ...................................................... : 108
MAM22 17 3 : ...................................................... : 108
MAM22_17_5 : ..........................C........................... : 108

              *       120         *       140         *       160
fragm17    : AATCTCACTAGCCCAtCTTCTCCCACAAGGAACACCTACATTCCTTATCCCTAT : 162
MAM22 17 2 : ...................................................... : 162
MAM22 17 3 : ...................................................... : 162
MAM22_17_5 : ...............C...................................... : 162

                    *       180         *       200         *
fragm17    : AATTATTATTATCGAAACCATTAGTCTCCTCATTCGACCAGTCACTCTAGCGGT : 216
MAM22 17 2 : ...................................................... : 216
MAM22 17 3 : ...................................................... : 216
MAM22_17_5 : ...................................................... : 216

              220         *       240         *       260         *
fragm17    : TCGACTCACCGCTAATATCACGGCAGGTCACTTACTTATTCATTTAACCGGAAC : 270
MAM22 17 2 : ...................................................... : 270
MAM22 17 3 : ...................................................... : 270
MAM22_17_5 : ...................................................... : 270

                    280         *       300         *       320
fragm17    : CGCCGCACTAACTTTACTAtCAATTCATTCAATAACAATTACAGTAACATTTAT : 324
MAM22 17 2 : ...................................................... : 324
MAM22 17 3 : ...................C.................................. : 324
MAM22_17_5 : ...................................................... : 324

                  *       340         *       360         *       3
fragm17    : TACTGTAgTTGTACTTACGATCCTGGAACTTGCcGTAGCACTAATTCAGGCCTA : 378
MAM22 17 2 : ...................................................... : 378
MAM22 17 3 : .......A.............................................. : 378
MAM22_17_5 : .................................T.................... : 378

             80         *       400         *       420         *
fragm17    : CGTCTTTGCCCTACTAATTAGCCTCTACCTACATGAGAGTGCTTAATGACTCAC : 432
MAM22 17 2 : ...................................................... : 432
MAM22 17 3 : ...................................................... : 432
MAM22_17_5 : ...................................................... : 432

                  440         *       460         *       480
fragm17    : CAAACACATGCCTATCACATAGTAGATCCAAGTCCCTGACCTCTTACCGGAGCA : 486
MAM22 17 2 : ...................................................... : 486
MAM22 17 3 : ...................................................... : 486
MAM22_17_5 : ...................................................... : 486

                *       500         *
fragm17    : TTATCTGCCCTACTTATAACATCT : 510
MAM22 17 2 : ........................ : 510
MAM22 17 3 : ........................ : 510
MAM22_17_5 : ........................ : 510
```
