# Supplementary material for: Complete Mitochondrial Genome and Phylogeny of Pleistocene MammothMammuthus primigenius
Source: PLoS Biol. 2006 Feb 7;4(3):e73. doi: 10.1371/journal.pbio.0040073 (PMC1360101; doi:10.1371/journal.pbio.0040073)
Supplement: Supplementary file 7 [file pbio.0040073.st001.doc]

**Table S1, page 1.**  Primer oligonucleotides and predicted PCR fragments used for sequencing of complete mitochondrial genome of *M. primigenius*, *E. maximus* and *L. africana*.

**Table S1 , page 2.**

Redundant primer oligonucleotides were used to amplify mammoth DNA. The sequences of mitochondrial genomes of *E. maximus* and *L. africana* were determined using both long-ranged PCR fragments and short PCR fragments.
