# Supplementary material for: Complete Mitochondrial Genome and Phylogeny of Pleistocene MammothMammuthus primigenius
Source: PLoS Biol. 2006 Feb 7;4(3):e73. doi: 10.1371/journal.pbio.0040073 (PMC1360101; doi:10.1371/journal.pbio.0040073)
Supplement: Supplementary file 8 [file pbio.0040073.st002.doc]

**Table S2.** Different types of substitutions revealed by pairwise comparisons of Elephantinae genomes.

|  | Mammoth-Asian | Asian-African | Mammoth-African |
| --- | --- | --- | --- |
| Transitions | 690 | 751 | 741 |
| Transversions | 32 | 43 | 39 |
| Synonymous | 529 | 555 | 559 |
| Nonsynonymous | 100 | 106 | 107 |

Number of synonymous and nonsynonymous substitutions calculated with the modified Nei-Gojobory method as implemented in MEGA [28], the results were independent of the transition/transversion ratio.
