# Supplementary material for: Complete Mitochondrial Genome and Phylogeny of Pleistocene MammothMammuthus primigenius
Source: PLoS Biol. 2006 Feb 7;4(3):e73. doi: 10.1371/journal.pbio.0040073 (PMC1360101; doi:10.1371/journal.pbio.0040073)
Supplement: Supplementary file 9 [file pbio.0040073.st003.doc]

**Table S3.** Baysean phylogeny inference [29] of Elephantinae using single mitochondrial protein coding and rRNA genes.

| Predicted sister species | Mammoth and Asian elephant | Mammoth and African elephant | Asian and African elephants | Star phylogeny of Elephantinae |
| --- | --- | --- | --- | --- |
| Protein and rRNA genes | 12SrRNA (0.72)  *ATP6* (0.68)  *COX1* (0.90)  *COX3* (0.92)  *CYTB* (0.76)  *ND1* (0.85)  *ND4L* (0.99)  *ND6* (0.96) | *COX2* (0.56)  *ND3* (0.92)  *ND5* (0.89) | 16SrRNA (0.88)  *ND2* (0.94)  *ND4* (1.00) | *ATP8* |

Posterior probabilities are shown in parentheses.
