# Supplementary material for: Complete Mitochondrial Genome and Phylogeny of Pleistocene MammothMammuthus primigenius
Source: PLoS Biol. 2006 Feb 7;4(3):e73. doi: 10.1371/journal.pbio.0040073 (PMC1360101; doi:10.1371/journal.pbio.0040073)
Supplement: Supplementary file 10 [file pbio.0040073.st004.doc]

**Table S4.** Lineage specific substitutions inferred by pairwise comparisons of Elephantinae genomes and polarized with one outgroup.

|  | Number of nonsynonymous substitutions in species I | Number of synonymous substitutions in species I | Number of nonsynonymous substitutions in species II | Number of synonymous substitutions in species II |
| --- | --- | --- | --- | --- |
| *M. primigenius* (I)   *E. maximus* (II) *L. africana* (outgroup) | 50 | 268 | 47 | 253 |
| *M. primigenius* (I) *L. africana* (II) *D. dugon* (outgroup) | 73 | 349 | 30 | 185 |
| *E. maximus* (I) *L. africana* (II) *D. dugon* (outgroup) | 78 | 356 | 26 | 181 |

We used the sequences of *E. maximus* and *L. africana* determined in this study for these comparisons. Pairwise comparisons of Elephantinae species using a single outgroup to polarize substitutions suggested that the *M. primigenius* – *E. maximus* clade evolves faster than the *L. africana* lineage in both synonymous and nonsynonymous sites.
