# Supplementary material for: Complete Mitochondrial Genome and Phylogeny of Pleistocene MammothMammuthus primigenius
Source: PLoS Biol. 2006 Feb 7;4(3):e73. doi: 10.1371/journal.pbio.0040073 (PMC1360101; doi:10.1371/journal.pbio.0040073)
Supplement: Supplementary file 11 [file pbio.0040073.st005.doc]

**Table S5.** Nucleotide diversity inferred by pairwise comparisons of different

|  | Number of SNPs | Number of nucleotides | Nucleotide diversity (π) |
| --- | --- | --- | --- |
| Two complete mitochondria (DQ316067 vs. NC_007596) | 48 | 16445 | 0.003 |
| Protein coding genes (DQ316067 vs. NC_007596) | 38 | 11316 | 0.003 |
| RNA genes (DQ316067 vs. NC_007596) | 4 | 4039 | 0.001 |
| CYTB (DQ316067 vs. D83047) | 6 | 1005 | 0.006 |
| CYTB (DQ316067 vs. D50842) | 5 | 1136 | 0.004 |
| CYTB (DQ316067 vs. NC_007596) | 5 | 1136 | 0.004 |
| 12S RNA (DQ316067 vs. D50841) | 0 | 961 | NA |
| 12S RNA (DQ316067 vs. NC_007596) | 1 | 961 | 0.001 |

*M. primigenius* sequences.

Sequences are reported in the following studies: DQ316067 (present study),

NC_007596 [20], D83047 [18] and D50841-D50842 [19].
