# Supplementary material for: Complete Mitochondrial Genome and Phylogeny of Pleistocene MammothMammuthus primigenius
Source: PLoS Biol. 2006 Feb 7;4(3):e73. doi: 10.1371/journal.pbio.0040073 (PMC1360101; doi:10.1371/journal.pbio.0040073)
Supplement: Supplementary file 12 [file pbio.0040073.st006.doc]

**Table S6.** Genome specific nonsynonymous and synonymous substitutions inferred by a pairwise comparisons of protein coding genes from two *M. primigenius* genomes (DQ316067 vs. NC_007596) and polarized with *E. maximus* (DQ316068) sequence.

|  | Nonsynonymous SNPs in NC_007596 genome | Synonymous SNPs in NC_007596 genome | Nonsynonymous SNPs in DQ316067 genome | Synonymous SNPs in DQ316067 genome |
| --- | --- | --- | --- | --- |
| All genes | 12 | 11 | 2 | 13 |
| *ATP6* | 0 | 0 | 0 | 0 |
| *ATP8* | 0 | 1 | 0 | 0 |
| *COX1* | 0 | 1 | 0 | 1 |
| *COX2* | 0 | 2 | 1 | 1 |
| *COX3* | 1 | 0 | 0 | 2 |
| *CYTB* | 0 | 2 | 0 | 3 |
| *ND1* | 2 | 1 | 0 | 0 |
| ***ND2*** | **7** | **2** | **0** | **1** |
| *ND3* | 0 | 1 | 1 | 1 |
| *ND4* | 1 | 0 | 0 | 2 |
| *ND4L* | 0 | 0 | 0 | 0 |
| *ND5* | 1 | 1 | 0 | 1 |
| *ND6* | 0 | 0 | 0 | 1 |

A large number of nonsynonymous substitutions was observed in the *ND2* gene (7 nonsynonymous versus 2 synonymous mutations in NC_007596 accumulated in a short ~360 bp region; whereas the DQ316067 sequence determined in this study had 0 nonsynonymous and 1 synonymous substitution in the same gene).
