# Supplementary material for: Complete Mitochondrial Genome and Phylogeny of Pleistocene MammothMammuthus primigenius
Source: PLoS Biol. 2006 Feb 7;4(3):e73. doi: 10.1371/journal.pbio.0040073 (PMC1360101; doi:10.1371/journal.pbio.0040073)
Supplement: Supplementary file 13 [file pbio.0040073.st007.doc]

**Table S7.** Genome specific substitutions inferred by a pairwise comparison of two *M. primigenius* genomes (DQ316067 vs. NC_007596) and polarized with *E. maximus* (DQ316068) sequence.

|  | NC_007596 mammoth genome | DQ316067  mammoth genome |
| --- | --- | --- |
| Total number of SNPs | 28 | 20 |
| (C->T and G->A) | (15 + 4) = 19 | (7 + 2) = 9 |

While the C->T and G->A substitutions may be a sign of ancient DNA decay (most frequent Type II mutations), they may also be real differences between the two genomes. The NC_007596 mammoth genome shows more of these substitutions, suggesting that a few of these substitutions may have occurred after death of the sampled mammoth, however, this difference is not statistically significant (Fisher’s exact test p > 0.05).
